# A scoping review of the conceptualisations of food justice

**DOI:** 10.1017/S1368980023000101

**Published:** 2023-04

**Authors:** Sandra Murray, Fred Gale, David Adams, Lisa Dalton

**Affiliations:** 1 School of Health Sciences, University of Tasmania, Newnham Drive, Launceston, 7250 Tasmania Australia; 2 School of Social Sciences, University of Tasmania, Launceston, Tasmania, Australia; 3 Tasmanian School of Business and Economics, University of Tasmania, Launceston, Tasmania, Australia

**Keywords:** Food justice, Food security, Social justice, Community participation and agency, Sustainable food systems

## Abstract

**Objective:**

The emerging concept of ‘food justice’ describes a social movement and a set of principles. It align with the goals of social justice, demanding recognition of human rights, equal opportunity, fair treatment and is participatory and community specific. The aim of this study was to investigate the conceptualisation of food justice and to explore how community participation is positioned in food justice scholarship.

**Design:**

A scoping review of peer-reviewed literature was conducted using the term ‘food justice’. This study used a five-step scoping review protocol. The databases included Scopus, Web of Science and Medline (OVID). Data were extracted on country of origin, research discipline, study type and conceptualisations of food justice. Reflexive thematic analysis was used to identify the themes.

**Results:**

The search identified 546 abstracts of which ninety peer-reviewed studies met the inclusion criteria. Thematic analysis identified five themes of food justice across these ninety studies: (1) social equity, (2) food security, (3) food systems transformation, (4) community participation and agency and (5) environmental sustainability.

**Conclusions:**

Current conceptualisations of food justice are evolving. Together, these five themes, using the term food justice, embrace a more holistic and structural view of the food system. They emphasise healthy, sustainable and equitable food as a human right and acknowledge the need to address structural barriers to that right. Community participation and agency in food justice decision-making is critical for transformative change towards a healthy, sustainable, and more just food system.

The emerging concept of ‘food justice’ describes a social movement and a set of principles. It aligns with the goals of social justice, demanding recognition of human rights, equal opportunity, fair treatment and is participatory and community-specific^(1,2)^. Its emergence also takes into account the global recognition that the food system is symbiotically linked to public health^(3)^. This is because of the connectivity that exists between food, health and the environment.

Worldwide farmers produce enough food to feed all citizens^(4)^. At the same time a combination of substantial food waste, inadequate food distribution and mass production of over-processed and low-nutritious food. Consequently, hunger is increasing^(5)^, the prevalence of undernourished people is escalating^(6)^, obesity is reaching epidemic proportions^(5)^ and public health being negatively impacted by escalating diet-related chronic disease rates. Furthermore, climate change influences agricultural production, and agricultural practices negatively impact the environment^(7)^. This has led to a ‘triple crisis’, whereby obesity, undernutrition and climate change undermine the conditions for human and planetary health^(8)^. Food justice is emerging as a powerful mobilising concept for driving social change to address food inequities from a more-than-human perspective^(9)^ – referring to the inseparability of human and natural interactions – but in theory and practice, it is a contested term^(10)^.

The world is at a critical juncture, as the state of global food insecurity – referring to a lack of access to sufficient and adequate food – increases after remaining virtually unchanged from 2014 to 2019 (FAO 2021). The United Nations Sustainable Development Goal (SDG) 2 ‘Zero Hunger’ is the human rights catalyst for governments to target food systems and environments to improve people’s access to safe, nutritious and sufficient food by 2030^(11)^. The 1948 Universal Declaration of Human Rights states ‘everyone has the right to a standard of living adequate for the health and wellbeing of himself and his family, including food…’. Although not legally binding, many countries endorsed a human rights-based approach to economic, social and cultural rights in 1975^(12)^. However, when it comes to equitable access to healthy, affordable and nutritious food, governments have predominately adopted a needs-based response, such as food relief, rather than a rights-based approach which is community-led^(13)^. The root causes of food insecurity are multifactorial and tend to include material hardships and inadequate financial resources^(14)^. The dominant response has focused on government welfare payments and supporting emergency food relief initiatives within the charitable food sector^(12,15)^. Such technical fixes are unlikely to solve food insecurity challenge^(16)^. These technical fixes are primarily focused on issues of food access, reflecting a ‘passive’ welfare ethos, locking people into welfare dependency^(17)^. While ‘community’ is understood as the site of the social problem, food insecurity will remain a site of political debate^(18)^.

The United Nations FAO has a vision for food security, which is when ‘… all people, at all times, have physical, social, and economic access to sufficient, safe, and nutritious food that meets their dietary needs and food preferences for an active and healthy life’^(19)^. Consistent with this vision, some governments (e.g. Australia) state that their national goal is to remain one of the most food security nations in the world^(20)^. National food security targets are often met by sourcing food produced under environmentally destructive and exploitative conditions and supported by subsidies and policies that destroy local food producers but benefit agribusiness corporations^(21,22)^.

Food system transformation – the radical change needed in our food system to dramatically improve environmental, health and livelihood outcomes – should be informed by timely and robust evidence. The literature on food security, social justice and the food system is extensive and varied, and continually evolving^(23)^. It ranges from assessments and proposed solutions to economic analyses, social and philosophical examinations of food as a human right and social justice issue^(24)^. To transform the food system, there is a call to broaden the scope of food security to go beyond the four well-documented pillars of availability, access, utilisation and stability to recognise sustainability and agency – where agency refers to the capacity of individuals and groups to exercise their voice and make decisions about their food systems – which are critical dimensions of food security and flow directly from the principles of the right to food legal framework^(23)^. The right to food, a legal right protected under human rights law, implies the right of people to feed themselves in dignity^(25)^. Sustainability seeks to achieve an environmentally sustainable food system^(26)^, which refers to how citizens can be empowered to exercise their capacity to make choices about what they eat, the foods they produce, how that food is produced, processed, distributed and their role in participating in policy processes that shape food systems^(23,27)^.

Agency is at the heart of community participation, which is critical for identifying an alternative future to the dominant food system, and advancing the social change needed to achieve transformation. Community-driven food system transformation, designed to facilitate human rights by promoting food security and addressing food insecurity, is simultaneously tackling the underlying structural determinants of food inequities and engaging in discourses and other local and global actions to confront these injustices^(28)^. At this juncture, the concept of food justice has emerged as a powerful mobilising concept for driving such social change.

Food justice has emerged as a response to social inequalities perpetuated by the mainstream food movement in the USA by marginalised communities seeking to address their food needs^(29,30)^. To understand what is required to progress food justice – that is, political platforms and policies – more needs to be known about the discursive contributions that the literature is making in this field.

In this study, we investigated the conceptualisation of food justice and explored how community participation is positioned in food justice scholarship. This paper is part of a larger project exploring the practice of food justice in two local communities in an Australian state.

## Methods

A scoping review was undertaken to examine the extent of the available evidence and highlight gaps in the existing research which explores food justice. A five-step scoping review protocol^(31)^ was used to (i) define the research question, (ii) identify the relevant studies, (iii) select the relevant studies, (iv) chart, collate and summarise the data and (v) report the results.

### Defining the research question

The aim of this review was to clarify the conceptualisation, use and practice of the term food justice. This review was guided by the following questions: What is the scope of publications on food justice according to country of origin, year of publication and discipline? How is food justice conceptualised with a particular focus on community participation? What is the frequency of research on food justice published in the peer-reviewed literature over time?

### Identification of relevant studies

A search of Medline (OVID), Scopus and Web of Sciences was conducted for all available studies preceding September 2020 using the term ‘food justice’ in publication titles and abstracts. The search used the following keywords and query strings for each database: ‘TITLE-ABS-KEY (food justice)’ in Scopus and ‘TS = food justice’ in the MEDLINE and Web of Science Core Collection. We did not restrict the search to specific publication dates or research areas. The reference lists of systematic reviews were searched to ensure that all relevant studies were included. Saturation was achieved when no new studies were identified.

### Selection of the relevant studies

All citations were imported to the EndNote™ reference manager and duplicates were removed. Using the inclusion and exclusion criteria (Table 1) citations, titles and abstracts were independently screened by two researchers one (SM) and two (LD). Discrepancies were resolved through consensus. Full texts of the included titles and abstracts were downloaded to Endnote citation software^(32)^ and read by both researchers. They were exported to the Covidence Review Software^(33)^, to manage the data screening process, whereby the two researchers undertook a full-text assessment for eligibility. Disagreements on full-text assessments were discussed by the researchers and resolved by consensus to eliminate selection bias.

**Table 1:** Inclusion and exclusion criteria

Studies were included in the review if they: • Described a conceptualisation of food justice rather than simply stated the term, where food justice was interpreted, practised or defined. • Published in English language • Were peer-reviewed qualitative, quantitative or mixed methods studies • Available online in full texts version • Human studies Studies were excluded in the review if they: • Did not meet the above criteria • Were opinion pieces, book chapters, conference abstracts, theses or grey literature.

### Charting the data

Key data that were extracted included study reference details (title, journal name, authors, discipline of the first author, year of study), study setting (country), study design, study rationale, research question, conceptualisations of food justice and primary outcomes relating to food justice. The disciplines of the first authors and their country of origin were extracted from the websites of their universities or institutes of affiliation and were used to identify the discipline from which the term or concept of food justice was interpreted. The data were exported from Covidence to a Microsoft Excel table to conduct the narrative synthesis.

This study required the identification and interpretation of patterns and themes within the data set; therefore, the Braun and Clark’s six-step thematic analysis process was used to determine the final food justice themes, including familiarisation, coding, iterative theme development, reviewing themes, defining and naming themes, and writing up^(34)^. Braun and Clark’s reflexive thematic analysis approach was applied^(35,36)^. The reflexive thematic analysis approach chosen for this study had a post-positivist approach to data analysis. This approach recognises the influence of researchers in interpreting data and encourages researchers to reflect on their influence as they develop and interpret codes^(34,35)^.

To ensure immersion, all selected literature was read by researcher one (SM) multiple times, coding all data, and researcher two (LD) double-coded 10 % of the studies^(37)^. A comprehensive list of key food justice terms for all included studies was extracted using an inductive approach to coding without the need for a coding framework (Table 3). These terms were analysed and grouped to reflect overarching themes and then labelled using the dominant recurring keyword representing the theme of that group. For example, terms such as food scarcity, food insecurity and hunger were grouped as like-terms, and the concept of food security was then used as a label to represent overarching themes. After one complete coding cycle, all codes and themes were documented, presented and discussed with the research team (SM, LD, DA and FG). The data were then uncategorised, re-coded, re-categorised, meaning checked and corroborated by researchers one and two. New codes and themes were identified during the iterative process. Themes were cross matched against the conceptualisations identified in each of the ninety studies (online Supplementary File 1). Major definitions were summarised to include the frequency of appearance of each term by study (Table 4), growth in the use of the term food justice by year and disciplinary origin (Table 5).

### Reporting the results

A description of the study selection process, study design, disciplines, years of publication and geographical distribution of the included studies as well as the conceptualisations of food justice were reported. Throughout this process, data were continually examined to make comparisons, examine contradictions and identify gaps, while keeping the aims of the paper at the front and centre^(35,37,38)^. The themes were discussed, reviewed, refined and checked by all the team members. Descriptions of some conceptualisations of food justice were insufficient, and interpretations of the terms were not always consistent. By adopting an approach based on conceptualisations of food justice, as well as definitions, the results provided a much broader understanding of how the term *food justice* was being used and adopted by authors. The results are described and presented as key themes of food justice. It was outside the scope of this review to report the methodological quality of the included studies.

## Results

**Fig. 1:**
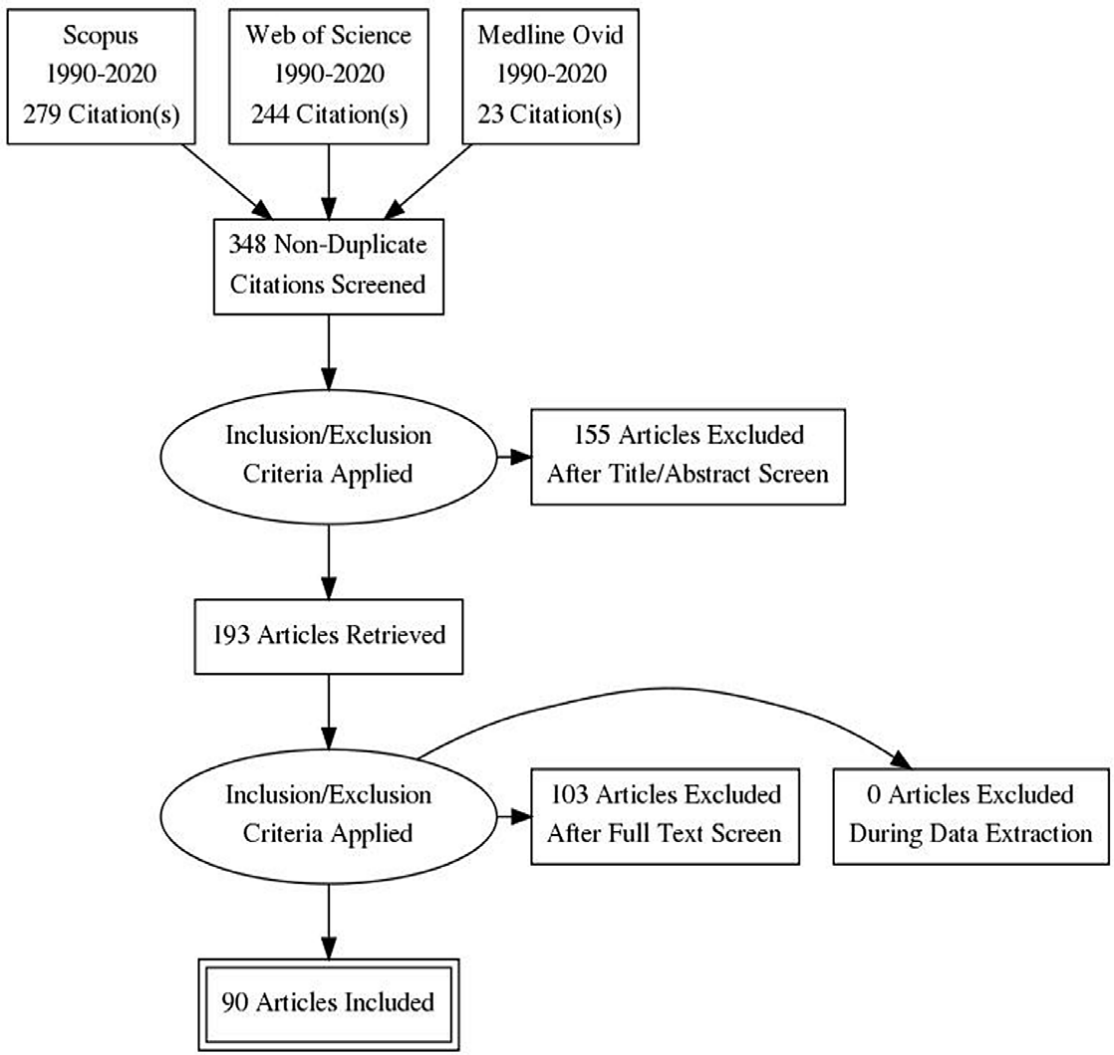
PRISMA flow diagram of search process

The search yielded 546 records from three databases: Medline (OVID), Scopus and Web of Science. Duplicates were excluded leaving 348 unique records for the analysis. Of the 348 records screened, 155 were excluded as irrelevant. The remaining 193 studies were subjected to a full-text assessment for eligibility, which led to 103 being determined as ineligible based on the inclusion/exclusion criteria in Table 1. The remaining ninety studies were included in the database for data extraction (Fig. 1).

### The scope of studies on food justice

Table 2 provides a descriptive summary of the identified studies on food justice. It identifies the scope of studies by country of origin, year of publication and discipline. Food justice was a highly interdisciplinary and expansive field of research that quadrupled each year after 2015, with most published in 2017. The highest concentration of studies came from high-income countries: the USA, the UK, Canada and Hong Kong. There was a complete absence of the term food justice in other geographical regions and from low- and middle-income countries. Ten disciplines clustered into three fields of knowledge^(39)^ published literature on food justice. The sociology and geography disciplines had the highest number of publications, representing 56 % of peer-reviewed studies and most were based in the USA and Canada. These studies have been associated with civil rights and social movements, the development of community-based approaches through alternative food practices, inequities in food access and environmental impacts. The methodologies were exclusively qualitative with case studies (61 %) examining existing initiatives, organisations and communities (Table 2).

**Table 2:** Descriptive summary of study characteristics of peer-reviewed publications

Country of origin	Year of publication	Study type	Disciplines
Canada (9) USA (71) UK (9) Hong Kong (1)	2004–2013 (12) 2014 (7) 2015 (16) 2016 (6) 2017 (17) 2018 (14) 2019 (7) 2020 (12	Case study (55) Theoretical (14) Review (9) Ethnographies (6) Commentary (3) Discourse or content analysis (3)	Social Science – Sociology (26), Geography (24), Political Science (5), Communication (3), Management (1), Law (1) Applied Science – Urban and Regional Planning (10), Environmental Education (8), Public Health (8), Humanities – Philosophy (4)

### Thematic analysis of the conceptualisations of food justice

Five themes of food justice conceptualisation were identified (Table 3). The themes and their frequency of appearance across the ninety publications (which were not mutually exclusive) were as follows: (1) social equity in 89 % of the studies, (2) food security in 79 %, (3) food systems transformation in 56 %, (4) community participation and agency in 66 % and (5) environmental sustainability in 39 %.

**Table 3:** Themes of food justice conceptualisations and frequency of appearance by study

Social equity (163)	Food security (156)	Food system transformation (157)	Community participation and agency (140)	Environmental sustainability (27)
Social injustice, exploitation (i.e. labour), oppression and exclusion (30)	Poor access to healthy affordable food (70)	Emergency food relief or food bank reform (10)	Participation, human rights, food systems activism and advocacy (31)	Climate (1)
Racism, class, gender, cultural politics (35)	Food poverty, apartheid, scarcity, hunger and unjust access to food (32)	Food system democratisation and redistribute power (80)	Self-reliance, empowerment and self-determination (4)	Sustainable practices (4)
Equity, rights, fairness, dignity and emancipation (23)	Food insecurity (23)	Food regimes, politicians and the state (2)	Planning for alternative food networks in urban and regional landscapes (8)	Human and environmental health (6)
Structural inequalities (7)	Foodways (2)	Policy solutions, building coalitions and reform (15)	Gentrification, loss of place and displacement (8)	Environmental sustainability (14)
Historical trauma, colonisation (6)	Food sovereignty (17)	Transformation. Tackling problems at local and national level (11)	Neoliberalism and the state *v*. non-commodified means of obtaining food (11)	Environmental/ecological justice movement (2)
Marginalised, low-income communities (33)	Community food security (12)	Food governance and policy (15)	Urban agriculture (30)	
Health inequities (26)	Food deserts (5)	Food policy council (6)	Activism, movements, resistance, direct-action, empowerment (27)	
Whiteness, white privilege (3)		Historical, holistic, distributive, participative procedural, recognition, justice and framing (18)	Community and school-led, owned and controlled food solutions (21)	

Within the five identified food justice themes, there were four themes including social equity, food security, food system transformation, and community participation and agency which all aligned with the core principles of social justice: equity, access, justice and rights^(24)^. Environmental sustainability, the fifth category aligned with the core principles of environmental justice, is the fair treatment and meaningful involvement of all people concerning the development, implementation and enforcement of environmental laws, regulations and policies^(40)^. Each of the five themes, while interconnected and broadly overlapping, reflect overarching central themes that are more powerful when explicitly stated. The themes and their alignment with social and environmental justice perspectives are discussed below presenting a deeper analysis to broaden the discussion of food justice.

Social equity, the largest category of studies, described persistent challenges to equity in the food system and the existence of structural inequality and discrimination. Terminologies such as structural inequality, exploitation, oppression, and social exclusion affecting marginalised and low-income communities were most prominent. Also prominent within this theme were terms such as racism, class, gender, cultural politics, white privilege, historical trauma and colonisation^(10,30,41–50)^. Health inequalities and disparities are also prominent^(43)^ with a focus on the intersecting issues of policy, health, social justice, economic development and the natural environment^(51,52)^. Such an emphasis suggests that food justice takes a broad social justice perspective and considers how unequal access to healthy food intersects with poor health^(53)^. There was a view that the voices of marginalised communities could be amplified by creating opportunities for dialogue with participants and improving health outcomes^(54–58)^.

Food security, the next largest category of studies, described inequitable access to healthy, affordable and culturally appropriate food and land. Terminology such as poor or unjust access to healthy and affordable food was most prominent, with terms such as food insecurity, food poverty, food apartheid, scarcity, hunger, right to food, food deserts, food sovereignty and community food security also used to imply challenges or solutions to accessing food^(28,29,43,44,47,54,56,59–72)^.

Food systems transformation was the third largest category of studies. These studies described dysfunctional food systems and called for structural and redistributive changes in the food system. Terminology included transformation, food systems democratisation, power, food bank reform, food policy councils, food governance and policy reform, tackling problems at a local and national level, policy solutions, building coalitions, politicians, and the state, justice (historical, holistic, participative, distributive, representational) and reframing^(10,44,45,49,55,61,65,73–83)^. The hope for food systems transformation focused on increasing food access and dismantling structural inequalities. It was claimed that^(84)^ it is insufficient for states to provide food relief without dismantling structural inequalities; therefore, food access is regarded as the entry point to food systems transformation and not the endpoint^(85)^.

Community participation and agency were the second least represented category and described community rights to participate and engage in food policy decision-making and governance, community resistance to and disruption of the existing corporate food regime and community-led, driven and/or owned food solutions. Terminologies such as participation, advocacy, self-reliance, self-determination, activism, movements and direct action were used to empower the voice of the community^(45,46,65,78,86–89)^. Also terminology such as alternative food networks, urban planning and non-commodification of the food system represented opportunities for the community to engage in food policy decision-making and governance^(44,56,75,79,82,90–93)^. Urban agriculture, community gardens, farmers’ markets, community and school-led, owned and controlled food solutions all provide opportunities for communities to be self-reliant, self-sufficient, empowered to take control and make decisions about their own food systems^(48,50,67,82,90,93–97)^.

Finally, environmental sustainability was the smallest category in the study. These studies described the need for food systems that minimise resource depletion and unacceptable environmental impacts. Terminologies such as climate change, human and environmental health, environmental sustainability and environmental justice were most prominent. While environmental concerns in the food justice literature were less prominent, their frequency of appearance increased from 2015^(10,28,43,44,51–54,59,60,65,71,73,77–79,83,90,98–102)^.

### Definitions of food justice

The major definitions of food justice were summarised, including the frequency of their appearance. Twelve definitions were identified, the two most frequently cited being Gottlieb and Joshi (developed in 2010 and expanded in 2013), and Alkon and Agyeman (developed in 2011). Gottlieb and Joshi’s^(103)^ definition was cited twenty-three times (including the original publication in which it appeared) and was reflected in four of the five identified themes (social equity, food security, food systems transformation and community participation and agency). Alkon and Agyeman’s^(29)^ definition was cited twenty-six times and reflected the same four food justice themes. The next most frequently cited definitions were those of Holt-Gimenez and Wang^(74)^ which was cited eleven times, Cadieux and Slocum^(44)^ cited ten times, Levkoe^(60)^ cited seven times and Hislop^(63)^ cited five times (Table 4). Based on an analysis of all twelve definitions, two distinct approaches to food justice were identified. The first, the ‘humanistic’ approach – referring to humans being placed front and centre – was reflected in ten definitions and was underpinned by the social justice principles of dignity, self-determination, equity, access, fairness and greater participation. The second ‘more-than-human’ approach was reflected in two definitions^(44,60)^ and combined social and environmental justice principles to include issues of distribution, participation and procedure, recognition and capabilities.

**Table 4:** Key definitions of food justice by author, year and frequency of appearance

Author	Year	Frequency of appearance	Approach	Definition
Lang & Heasman	2004	2	Humanistic	‘…the maldistribution of food, poor access to a good diet, inequities in the labour process and unfair returns for key suppliers along the food chain’
Levkoe	2006	7	More-than-human	‘…promote a strategy of food security where all people have access to adequate amounts of safe, nutritious, culturally appropriate food produced in an environmentally sustainable way and provided in a manner that promotes human dignity’
Ahmadi	2007	1	Humanistic	‘… no one should live without enough food because of economic constraints or social inequalities… The food justice movement is a different approach to a community’s needs that seeks to truly advance self-reliance and social justice by placing communities in leadership of their own solutions and providing them with the tools to address the disparities within our food systems and within society at large’
Alkon & Norgaard	2009	7	Humanistic	‘… places the need for food security—access to healthy, affordable, culturally appropriate food—in the contexts of institutional racism, racial formation, and racialised geographies’
Gottlieb & Joshi	2010 (modified in 2013)	23	Humanistic	‘a social movement with multiple layers… ensuring that the benefits and risks of where, what, and how food is grown and produced, transported, and distributed, and accessed and eaten are shared fairly… Three Arenas of food justice (1) seeking to challenge and restructure the dominant food system, (2) providing a core focus on equity and disparities and the struggles by those who are most vulnerable, (3) establishing linkages and common goals with other forms of social justice activism and advocacy’
Holt-Gimenez & Wang	2011	11	Humanistic	‘… tends to be more progressive than reformist in that it addresses specifically the ways in which people of colour in low-income communities are disproportionately and negatively impacted by the industrial food system’
Alkon & Agyeman	2011	26	Humanistic	‘… an analysis that recognises the food system itself as a racial project and problematises the influence of race and class on production, distribution, and consumption of food. Communities of colour and poor communities have time and time again been denied access to the means of food production, and, due to both price and store location, often cannot access the diet advocated by the food movement. Through food justice activism, low-income communities and communities of colour seek to create local food systems that meet their own food needs’
Sbicca	2012	4	Humanistic	‘… a budding social movement premised on ideologies that critique the structural oppression responsible for many injustices throughout the agri-food system’
Loo	2014	1	Humanistic	‘Present definitions tend to conceptualise food justice in distributive terms as being a matter of improving wages and conditions for those working in the food system and ensuring and the fairness in which fresh and healthy food is distributed amongst eaters……. participative disparities are at the root of the most important distributional disparities. Even those who endorse distributive accounts of food justice tend to argue for interventions that are in essence strategies to improve the ability of vulnerable individuals to participate in decision-making and the governing of food systems. One way to start thinking about how fair participation is to be achieved is to think about what minimally is required for individuals and communities to consent to decisions or activities that may affect their food system. Food justice scholarship and the food justice movement must be more careful to consider the importance of participation in decision-making regarding distributive inequalities’
Hislop	2014	5	Humanistic	‘…the struggle against racism, exploitation, and oppression taking place within the food system that addresses inequality’s root causes both within and beyond the food chain’
Cadieux & Slocum	2015	10	More-than-human	‘Four nodes of food justice include 1) Trauma and equity - acknowledging and confronting historical, collective social trauma and persistent race, gender, and class inequalities; 2) Exchange - designing exchange mechanisms that build communal reliance and control; 3) Land - creating innovative ways to control, use, share, own, manage and conceive of land, and ecologies in general, that place them outside the speculative market and the rationale of extraction; and 4) Labour - pursuing labour relations that guarantee a minimum income and are neither alienating nor dependent on (unpaid) social reproduction by women’
Horst	2017	3	Humanistic	‘… advocates for dismantling institutional racism and policies and programs that support inequalities in the food system’

### The frequency of research on food justice over time

The use of the term food justice has grown since 2004, with an expansion in the five identified food justice themes from 2015 (Table 5). While all themes have increased in prominence, there has been a specific upward trend in themes addressing food systems transformation, community participation and agency, and environmental sustainability.

**Table 5:** Growth in food justice literature by year, discipline cluster and category

Years	Discipline	Social equity	Food security	Food system transformation	Community participation and agency	Environmental sustainability
2004	AS	1	1	1	1	1
2005						
2006	AS	1	1		1	1
2006						
2008	SS	1				
2009	SS	1	1		1	1
2010	AS, SS	2	2	2	4	1
2011	PH	1	1		1	
2012	AS, SS	2	2	1	4	1
2013	SS	2	1	1	2	1
2014	H, SS	6	4	6	5	
2015	AS, SS, PH	12	11	9	10	6
2016	AS, SS	5	4	4	5	4
2017	AS, SS, PH	15	15	10	19	8
2018	AS, SS	14	15	5	11	4
2019	AS, SS	7	7	2	2	1
2020	AS, SS	11	7	8	9	5

AS, Applied Science; SS, Social Science; H, Humanities; PH, Public Health.

## Discussion

This scoping review found that, since 2004, the majority (77 %) of food justice studies, using the term, quadrupled each year from 2015 with 2017 being the most prolific publication year. The spike in publications can be linked to the momentum fostered by the official adoption of the UN SDG in late 2015^(11)^, the concurrent general rise in food studies scholarship and the ‘social justice turn’ – adoption of an anti-oppressive stance – in the broader social and environmental literature. Publications on food justice came from the USA, Canada, the UK and Hong Kong (Tables 2 and 5). This distribution represents a long history of social justice activism in these countries that has been led by marginalised groups tackling inequality, poverty and injustice, in which food has often played an important role^(44,104)^. The limitation of this distribution does not mean that social justice activism for food system transformation has not occurred in other countries. Conversely, this may mean that relevant literature in other languages has not been included due to the exclusion of non-English language literature in our exclusion criteria.

The multidisciplinary body of food justice research reported in this scoping review incorporates contributions from ten disciplines organised into three academic clusters or fields of knowledge. Within the social sciences publications, the discipline of sociology (47 %) was the most prolific, followed by geography (44 %) within the applied sciences, and public health (including nutrition) (9 %). From over 40 000 journals searched, 348 papers were reviewed, and several academic disciplines were not identified or had limited presence, including agricultural science, economics, management, law and philosophy. These disciplines are active and influential in food policy debates and discussions related to food security; curiously, they have yet to engage with the food justice debate. Conversely, these disciplines may have engaged and been published in different journals or formats, such as essays, rather than peer-reviewed journals. Further research is required to understand the reasons for this.

Since 2015, there has been a shift in the conceptualisations of food justice. The five themes identified in this review may be attributable to a movement towards challenging and restructuring the dominant food system in-line with global expectations to deliver progress on all seventeen SDG^(105)^. The underlying premise of the SDG vision rests on principles of human rights and social justice that emphasise dignity, self-determination, equity, access, fairness, and greater participation^(106)^, and principles of environmental justice that emphasise distribution, participation, and procedure, recognition, and capabilities^(107)^.

Challenging and calling for the dominant food system to be restructured may also arise from a tendency to link the construct of food justice with other forms of social justice, such as activism and advocacy^(10)^. More recently, published literature indicates the need for further community participation and agency. While such actions align with human rights-based approaches to food security^(108)^, this alone is unlikely to change policy, systems and practice. An integrated food systems approach requires a more-than-human rights-based approach that implies the inseparability of human and natural interactions and the right of people to feed themselves with dignity^(9,25)^.

### Broadening the view of food justice

The scoping review identified that food justice has been conceptualised mostly in distributional terms in the definitions of Lang and Heasman^(109)^, Levkoe^(60)^, Alkon and Norgaard^(54)^, Gottlieb and Joshi^(103)^, Holt-Gimenez and Wang^(74)^, Hislop^(63)^, Cadieux and Slocum^(44)^ and Horst^(46)^ (Table 4). These conceptualisations reflect the elements of the World Food Summit’s (1996) definition of food security, which was further refined in the FAO 2002 definition given at the beginning of this study. At its core, the FAO definition embraces a social justice approach using distributive justice – the fair allocation of resources – as a mechanism to get food to people who lack access. In practice, this has a range of positive and pernicious effects. The focus on food distribution responsibilises individuals and households to meet their own food security needs in the first instance and encourages interventions such as emergency food relief practices in specific instances when individuals and households fail to do so^(15)^. This system ‘feeds on itself’, as emergency food relief distribution practices then enable the structural factors underpinning the original inability of the household to meet its food security needs – lack of financial resources, failed land reform, poor food quality, low levels of community empowerment and food inappropriateness – to be downplayed or ignored.

This scoping review also found that food justice was increasingly conceptualised in procedural terms. Procedural justice moves beyond distribution and centres on participation in the decision-making process or policies used to make allocation decisions and manage resources, suggesting that participative disparities are at the root of the most important distributional disparities^(110)^. This approach challenges the structural accounts of people being at the mercy of law, politics and economics. Instead, people are repositioned as active agents capable of ascribing freedom and responsibility and facilitating change. This literature considers humanistic procedural justice emancipatory because it offers less powerful stakeholders a mechanism to challenge the corporatisation of food systems and the harmful technological fixes that are considered detrimental to the environment.

An example of this procedural emancipatory justice approach is the Global People’s Summit on Food Systems^(111)^, which was launched as a counter-summit to the UN Food Systems Summit in 2021^(105)^. The Global People’s Summit drew worldwide attention to the vulnerabilities and lack of sustainability inherent in the current global commodified export-based food system^(112)^. However, in the food justice literature, the Summit’s identified social and environmental concerns, under the broad concept of ‘food sovereignty’, are not currently heavily weighted in the dominant conceptions of food justice identified in this scoping review. Food sovereignty embraces a linked social–economic–environmental conception of justice and views local food systems as the most appropriate way to tackle sustainable food security. It responsibilises communities (local, state and national governments) by ensuring food security, which is understood as a fundamental political and economic right, and seeks to empower those who have failed by their communities to take affirmative action^(113)^. This scoping review argues that distributional inequalities often result from participatory inequalities^(114,115)^.

### An emerging opportunity for community participation and agency

This review supports the view of many food justice theorists^(2,44,116–118)^ of a paradigmatic shift in the conceptualisation of food justice over the past two decades. The concept of food justice has expanded beyond its initial relatively limited meanings predominately underpinned by the social justice principles of the need to respond to food insecurity. Food justice now references multiple justice themes, including procedural, emancipatory and humanistic conceptions that obligate governments (i.e. local, state and national governments) to be primarily responsible for ensuring food security as a fundamental political-economic right. The approach is linked to enabling local communities to achieve food justice through empowerment which enables participation and respects agency^(27)^. Local action by people with lived experience of food insecurity plays a vital role in addressing the root causes of food injustices throughout the food system and ensuring that society works towards food justice for all^(118)^. Furthermore, the concept of food justice acknowledges and welcomes ‘other actors’ into the food system, as these are required to address various structural barriers to ensure that the rhetoric of food justice becomes a reality.

If governments and communities can work together, there are new opportunities to use food justice and rights language when conveying food security information; embrace grass roots advocacy to raise awareness and contextualised understandings of the issue; create dialogue among community participants to advocate fair food policies that affect them; develop community leaders to be experts in healthy and sustainable food systems; provide resources that community participants need to succeed in such as policy-relevant scientific evidence; bring the voices of underrepresented groups into the conversation and provide resources and skills. At the policy level, opportunities exist for evaluating policy changes that document benefits and limitations, and integrated food justice and rights into government frameworks and community projects^(12,55)^.

While different stakeholders are likely to continue emphasising the specific themes of food justice to achieve different outcomes, it is useful to place this in a broader context. For example, some groups aiming to improve food justice outcomes in local municipal regions, where race and ethnic issues are central defining features, are likely to place greater emphasis on social equity. For others who seek to prevent obesity and diabetes and respond to the rapidly increasing rates of chronic disease, high-level policy reform linked to media advertising, food literacy and sugar taxes will come into focus. However, regardless of the specific focus, stakeholders must place their food justice conceptions within a broader consideration of the structural and environmental factors in play. Urbanisation, wage stagnation and the impacts of COVID-19 on the one hand and climate change, deforestation and biodiversity loss must be considered in any discussion of the food system and its link to social issues, such as public health.

Definitions of food justice vary, which means that the practice of food justice could be challenging to execute^(98)^. The interpretation of justice as it relates to food production, distribution and consumption is multifaceted and complex^(119)^. Perhaps the fragmented nature of food justice is its strength, with its potential to be flexible in implementing local solutions to global issues^(59)^. Food justice is only now beginning to mature to embrace a holistic and structural view of the food system that understands healthy food as a human right and acknowledges the need to address various structural barriers to that right^(10,120)^. Community participation, agency, activism and empowerment must be part of any food justice plan for self-sufficiency and sustainability.

### Limitation

This review had three limitations. First, it limited the search term to ‘food justice’ and Boolean strings such as ‘food AND justice’ were not applied. The search strategy, therefore, did not encompass all terms related to ‘food justice’ such as the right to food, food sovereignty, food security or food democracy studies. This enabled the authors to keep the study closely focused on the concept of ‘food justice’ to generate a manageable database. Second, the search was limited to three databases which, although larger, were not comprehensive. Taken together, these limitations likely overlooked relevant studies. Despite these limitations, the search strategies yielded many studies in which ‘food justice’ *and* related terms were the focus of the review, and the existence of considerable duplication across the databases indicates that the studies were drawn from a larger, shared sample. Third, there is an opportunity for further development of the five food justice themes, given their close interlinkage. Future reviews could encompass other conceptualisations and the connections between them and additional databases, to draw a wider picture of the relationships between marginalised communities and food injustice.

## Conclusion

Despite its almost 20-year history, the parameters of food justice continue to evolve, with limited consensus in the literature on what food justice is, making it difficult for communities to mobilise for transformative food system changes. Food justice is a multidisciplinary concept that is rooted in human rights and justice. Most commentators embrace a ‘humanistic’ position to conceptualise and define food justice by drawing on the principles of social justice. This discursive position is likely to sustain food banks and food relief approaches to food security. A less prominent ‘more-than-human’ conception of food justice has recently emerged to combine social and environmental justice principles in a quest for human well-being and global sustainability. Combining the social justice principles of dignity, self-determination, equity, access and participation with environmental justice principles that emphasise participation, distribution and procedure, recognition and capabilities enables a broader and more contemporary conception of food justice. The broader conceptualisation of food justice can be used as a powerful mobilising concept for a range of stakeholders to contribute to food system transformation discussions and planning by advocating the health benefits of food justice. Furthermore, it can inform calls for a more integrated approach to healthier and more just local and national food policies, systems and practices that address obesity, undernutrition, climate change and other environmental issues that decimate human and planetary health.

Community participation and agency in food justice decision-making is critical for transformative change towards a healthy, sustainable and more just food system.

## Supplementary material

For supplementary material accompanying this paper visit https://doi.org/10.1017/S1368980023000101. click here to view supplementary material
