# Supplementary material for: A scoping review of the conceptualisations of food justice
Source: Public Health Nutr. 2023 Jan 23;26(4):725–37. doi: 10.1017/S1368980023000101 (PMC10131151; doi:10.1017/S1368980023000101)
Supplement: Supplementary file 1 [file S1368980023000101sup001.docx]

**Supplementary File 1 – Summary of Food Justice conceptualisations by author, year, and category.**

| **Authors** | **Year** | **Conceptualisation (definition or component parts)** | **Social Equity** | **Food security** | **Food system transformation** | **Community participation and agency** | **Environmental sustainability** |
| --- | --- | --- | --- | --- | --- | --- | --- |
| Wekerle G | 2004 | Transition to a focus on the right to food as a component of a more democratic and just society and, most recently, a reframing as food justice movements. The food justice frame highlights the focus on systemic change and the necessity for engaging in political and policy processes as well as consciously addressing issues of movement mobilization and strategies. Theoretically, the food justice frame opens linkages to a wider range of conceptual frameworks drawn from the literature on democracy, citizenship, social movements, and social and environmental justice. | X | X | X | X | X |
| Levkoe C | 2006 | Food justice movements promote a strategy of food security where all people have access to adequate amounts of safe, nutritious, culturally appropriate food produced in an environmentally sustainable way and provided in a manner that promotes human dignity | X | X |  | X | X |
| Guthman J | 2008 | Current activism reflects white desires more than those of the communities they putatively serve. conclude that more attention to the cultural politics of alternative food might enable whites to be more effective allies in anti-racist struggles. | X |  |  |  |  |
| Alkon A, Norgaard K | 2009 | Places access to healthy, affordable, culturally appropriate food in the contexts of institutional racism, racial formation, and racialized geographies. Serves as a theoretical and political bridge between scholarship and activism on sustainable agriculture, food insecurity, and environmental justice. | X | X |  | X | X |
| Bedore, M | 2010 | Food justice model to forge new theoretical linkages to decommodification, democracy, citizenship, social movements, and environmental justice (Huish 2008; Johnston et al. 2009; Wekerle 2004). Useful for looking beyond supply oriented approaches or food charity models often employed by anti-hunger or food security models (Power, 1998). | X | X | X | X | X |
| Hughes L | 2010 | food justice lens is that it situates of food security, right to food and food sovereignty discourses and practices within multiple and interlocking systems of inequality across lines of differentiation such as race, class, and gender, as well as across spatial scales. Food justice movement seeks to truly advance self-reliance and social justice by placing communities in leadership of their own solutions and providing them with the tools to address the disparities within our food systems and within society at large (Ahmadi 2007). | X | X | X | X |  |
| Freudenberg N, McDonough J, Tsui E | 2011 | Public health history suggests that strong movements can play an essential role in achieving the transformation necessary to make healthy and affordable food available to all. Public health professionals can amplify the health effects of such movements by creating opportunities for dialog with movement participants, providing resources such as policy-relevant scientific evidence, documenting problems, and evaluating policies, and offering technical, political, and organizational development expertise. | X | X |  | X |  |
| Alkon A, Mares T | 2012 | The concept of food justice speaks to the multiple ways that racial and economic inequalities are embedded within the production, distribution, and consumption of food. Activists working from these two perspectives often create local food system alternatives such as farmers markets, CSA programs, urban farms, and cooperatively owned grocery stores in low-income communities of colour. Additionally, food justice activists tend to emphasize the need for these projects to be created not only for, but by members of these communities. | X | X |  | X |  |
| Sbicca J | 2012 | Action for food change takes many forms, from street protests to the quiet sustainability of community gardening, to advocacy and acting for food policy change Kneafsey, Owen et al. (p. 622) explores the quiet radicalism of food growing mentorship programmes: we interpret them as examples of capacity building for food justice, through quiet steps rather than radical transformation. |  |  | X | X | X |
| Heynan N, Kurtz H, Trauger A | 2012 | Working to better link community food security, food sovereignty and urban agriculture because not only are they historically and theoretically robust, they also have resulted from organizing struggles in food insecure scenarios and all have shown productive possibilities for addressing hunger. | X | X |  |  |  |
| Kato Y, | 2013 | The food justice movement has emerged partially in response to criticisms of the alternative food movement and aims to directly engage the social justice issues. The movement is multifaceted, with varying and sometimes conflicting interpretations of justice as related to food production, distribution, and consumption (Anderson 2008). Wekerle (2004) argues that the fragmented appearance of the movement is its strength, for its broad network bridges local solutions to global issues, but recent academic writing on the food justice movement has taken a more critical approach in examining different interpretations of justice among activists and scholars (e.g. Allen 2010; Bedore 2010; DuPuis, Harrison, and Goodman 2011). Conflicting definitions of food justice could pose challenges in their execution in practice as each interpretation of food justice may call for distinct solutions. Despite sharing the conceptualization of justice grounded in a civil rights framework (Gottlieb and Fisher 1996), the food justice movement has not fully taken advantage of developments in the environmental justice movement. | X |  | X | X | X |
| Gibb N, Wittman H | 2013 | Gottlieb and Joshi (2010) characterise food justice as ensuring that the benefits and risks of where, what, and how food is grown and produced, transported, and distributed, and accessed and eaten are shared fairly (p. 6). Food justice involves ensuring that food system benefits and burdens are shared fairly; ensuring equal opportunities to participate in food system governance and decision-making; and ensuring that diverse perspectives and ways of knowing about the food system are recognised and respected. | X | X | X |  |  |
| Purifoy D, | 2014 | Food justice is equitable access not only to healthy, culturally appropriate food, but also to the benefits of food production and distribution for all communities. (Gottlieb and Joshi 2010). | X | X |  |  |  |
| Passidoma C | 2014 | All effective justice-oriented food projects must confront both the chronic and acute injustices that circumscribe food access and constrain food sovereignty; otherwise, they risk reinforcing and exacerbating the very conditions that have generated the dominant and unjust contemporary food regime these initiatives purport to undermine. | X | X | X |  |  |
| Agyeman J, McEntee J | 2014 | Food justice as a social movement arose largely from urban-located social justice groups that explicitly addressed food inequalities based on race and/or socioeconomics. Addressing the causes of inequality in the conventional agri-food system and making justice considerations explicit because race and class play a central role in organizing the production, distribution, and consumption of food (Alkon and Agyeman 2011, p.4). | X | X | X | X |  |
| Bradley K, Galt R | 2014 | Food justice embraces a variety of ways of valuing food system work, including self-improvement and community improvement, and need not, though may, include learning about nutrition, health, and local or organic food (White 2011). Self-determination at the organisational and individual levels and respect for others are crucial to food justice practice. In the food justice context, we understand self-determination as control of the means of production in a local food system and influence in the social arrangements shaping pro-duction and exchange. | X |  | X | X |  |
| Loo C | 2014 | The definitions of food justice currently being offered by scholars and activists omit important aspects of the disparities inherent to food systems. Present definitions tend to conceptualize food justice in distributive terms as being a matter of improving wages and conditions for those working in the food system and ensuring and the fairness in which fresh and healthy food is distributed amongst eaters. Food justice involves stakeholders being: (1) informed of the likely benefits and risks of decisions affecting their food system, (2) provided with sufficient information to be able to understand the implications of those risks and benefits, (3) adequately educated so that they are competent to make decisions, and (4) able to assent or dissent from decisions without coercion. | X | X | X |  |  |
| Dixon B | 2014 | distinguish the aims and goals of food justice advocates from those who critique the dominant food system and identify an alternative approach (Gottlieb and Joshi 2010, p. 6). Food justice narratives are forward looking as well because they bring into clearer focus what actions and kinds of social activism are appropriate responses to constraints on free choice. | X |  | X | X |  |
| Werkheiser I, Noll S | 2014 | We argue that most of the critiques levelled against local food are directed against the individual-focused sub-movement, which is most compatible with the current industrial food system, and perhaps not surprisingly receives the most mainstream attention. Further, we argue that while each movement has its own strengths and weaknesses, it is the community-focused sub movement that has the most potential to radically transform the global food system. |  |  | X | X |  |
| Brent Z, Schiavoni C, Alonso-Fradejas A | 2015 | food justice is communities exercising their right to grow, sell, and eat [food that is] fresh, nutritious, affordable, culturally appropriate, and grown locally with care for the well-being of the land, workers, and animals (Alkon, Agyeman, 2011). However, within this frame we find an array of perspectives that highlight the tensions identified by Holt-Gimenez and Shattuck (2011), ranging from progressive approaches, where the priority is increasing food access without necessarily dismantling structures causing inequality, to radical approaches that indeed seek to dismantle such structures and see food access as an entry point. | X | X | X | X |  |
| Anguelovski, I | 2015 | The right to healthy, fresh, local, and affordable food for com-munity food security is one of the focus points of community advocacy for urban food justice (Alkon and Agyeman, 2011; Gottlieb and Joshi, 2010; Hess, 2009). |  | X |  | X |  |
| Clendenning J, Dressler W, Richards C | 2015 | Food justice grew out of racial inequalities, and was initially designed to promote food access, leading to more distributive food movements. Food justice movement seeks to address injustices that disproportionately impact upon people based on race and class (Gottlieb and Joshi 2010; Mares and Alkon 2012). Food justice [rather than food sovereignty] has spread in the US, because they expect the state to give them that. I think this is the fundamental ideological piece of why food sovereignty doesn't resonate in the US. | X | X | X |  |  |
| Sbicca J | 2015 | Gottlieb and Joshi (2010, p. 38) argue, that the centre of the food justice ethos is the demand for justice in the fields and workplaces that produce and process food, and for recognition of the dignity of work and basic human rights for those who have been denied such rights. Alkon and Agyeman (2011, p. 339) conclude that food justice activists might find allies who understand the relationship between food and racial and economic exploitation from a different angle and could offer additional visions of what a just and sustainable agriculture might look like. Labour issues are central to food justice and to assembling eaters, workers, scholars, and policymakers (Holt-Gimenez, 2011). Food justice requires creating economic opportunities for the most marginalized (Sbicco 2015) | X | X | X | X | X |
| Kaiser M, Himmelheber S, Miller S, Hayward A | 2015 | Contemporary food justice work focuses on the intersecting issues of policy, health, social justice, economic development, and the natural environment. Food justice work is environmental justice work. | X | X | X | X | X |
| Kepkiewicz L, Chrobok M, Whetung M, Cahuas M, Gill J, Walker S, Wakefield S | 2015 | Food justice initiatives can be problematically rooted in white desire to enrol black people in a particular set of food practices (2008, p. 433). When food justice involves improving and providing charity it rarely addresses [es] the source of inequality... bringing individual improvement rather than allowing for (or supporting) collective action (Guthman 2011, p. 157). | X | X |  | X |  |
| Slocum R, Cadieux K | 2015 | Food justice activism is not only the purview of people of colour. That said, without acknowledging the history of antiracist activism in the food movement and its relationship to today's food justice mobilizing, scholars might be inclined to forget that the food movement cannot be studied without asking "what does race do here?" (Slocum and Saldanha 2013). Accountability means acknowledging the history of antiracist activism in the movement and its relationship to what is now called food justice. Although the association had been made between environmental justice and food by key food movement organizers (Gottlieb and Fisher 1996), it was not until the 2003 Boston Community Food Security Coalition (CFSC) conference that an organized and collective antiracist agenda within this part of the food movement gathered strength. | X | X |  | X | X |
| Del Casino V. | 2015 | Food justice movements concern themselves with more than the landscapes of retail consumption. Instead, food justice scholars suggest a move toward food sovereignty and community food security. Attempts to shift focus away from a reductionist food desert paradigm can be seen in the development of scholarship on both food security and food justice (e.g., Miewald and McCann, 2014) |  | X |  |  |  |
| Burke J, Spiller K | 2015 | explore efforts and commitments that explicitly focus on the role of racial equity and food justice as integral components of food system transformation. As we challenge the status quo, share strategies, and explore policies and practices designed to promote greater racial equity and food justice, we realize this is indeed a journey in which we are all learning. We have a glimpse of the possibility of a just and healthy food system. To get there, we must use a critical race lens to diagnose what€™s wrong with the current system, assess entry points for change, and determine ways that we can work together to build a better system for all of us. (Gaincatarino & Noor, 2014, p. 4). | X |  | X |  |  |
| Hayes C, Carbone E | 2015 | Food justice encompasses a vision of social, environmental, and economic justice; improved nutrition and health; and community activism [2]. While different disciplines tend to define it through their own respective lenses, food justice is a concept that informs multiple areas of thought, action, and study, including: 1) alternative, activist, and oppositional food movements [4,7,9-13] 2)environmental justice [2,4,11,14,15] 3)food security [2,6,7,12,13] 4)health equity and disparity [7,11,13,16] . As Gottlieb and Joshi explain, 'What connects these approaches is the desire to create fundamental change as well as alternatives to the dominant food system'. | X | X | X | X | X |
| Vitiello D, Grisso J, Whiteside K, Fischman R | 2015 | Hamm and Bellows classic definition of community food security as a condition in which all community residents obtain a safe, culturally acceptable, nutritionally adequate diet through a sustainable food system that maximizes community self-reliance, social justice, and democratic decision-making (2003: 37). | X | X | X | X | X |
| Dobson J | 2015 | Food security has been defined by the U N Food and Agriculture Organisation as: when all people, always, have physical and economic access to sufficient, safe, and nutritious food to meet their dietary needs and food preferences for an active and healthy life. The notion of food justice specifically acknowledges that people do not have fair access to safe and nutritious food and that the production, retailing and consumption of food exacerbates inequalities. (Levkoe, C. Z. 2006). These inequities are experienced at global and local levels. | X | X |  |  |  |
| Miller W | 2015 | The literature on food justice movements (Wekerle 2004, Levkoe 2006) suggests that diverse food projects in urban areas represent a reclaiming of urban space. |  |  |  | X |  |
| Cadieux K, Slocum R | 2015 | Food justice represents "a transformation of the current food system, including but not limited to eliminating disparities and inequities"(Gottlieb and Joshi 2010: ix). Food justice means transformative change at four key points of intervention: trauma/inequity, exchange, land, and labour. (Cadieux & Slocum 2015). aims to distinguish between an industrial food system and a more equitable, ecologically viable alternative (Alkon, Agyeman, 2011). | X | X | X |  | X |
| Vernon R | 2015 | Too often, those involved in food justice work see the lack of consumption of healthy foods as an issue of personal choice rather than one resulting from a deeply traumatic history of food relations in Native communities. | X |  |  |  | X |
| Sbicca J | 2016 | Food justice strives to eliminate and challenge social inequities within and beyond the food system. Sbicca 2016. Food justice advocates for the right to healthy food that is produced justly and sustainably, recognizes diverse cultural foodways and histories, promotes democratic participation and control over local food systems, and equitable distribution of resources in the food system (Alkon and Agyeman 2011; Cadieux and Slocum 2015; Gottlieb and Joshi 2010; Hislop 2015). | X | X | X | X | X |
| Agnelli K, Cramer E, Buffington M, Norris J, Meeken L | 2016 | Literature about food justice interventions suggests a few typologies of intervention: community gardens and farmers markets (Kato,2013; Widener, Metcalf, & Bar-Yam,2012), local supermarket development and convenience store inventory changes (Song et al.,2009; Walker, 2010), and policy change initiatives (Lappe, 2009). |  |  | X | X |  |
| Bradley K, Herrera H | 2016 | The food justice movement is fundamentally a social justice movement. The notion of food justice struggles against racism, exploitation, and oppression taking place within the food system (Hislop 2014) are integrated with practical efforts to establish fair, equitable access to fresh, healthy, affordable, culturally appropriate food in vulnerable neighbourhoods, especially low-income neighbourhoods and communities of colour, with ownership and governance of the means of production and exchange accessible to the people who eat this healthy food. It takes issue with inequalities in access to food, exploitive labour practices in the food system, and environmental degradation associated with conventional agriculture and environmental racism (Alkon and Agyeman 2011; Gottlieb and Joshi 2010) | X | X | X | X | X |
| Shannon D | 2016 | FNB actively argue for food access as a basic right in their slogan 'food is a right, not a privilege'. activists argue that basic human rights and dignity might be met through the direct action of affected populations rather than relying on states, politicians, and/or legislative bodies to meet our collective needs. According to Food Not Bombs activists, this orientation is central to human liberation. Rights discourse is enforced and maintained by the powerful and an appeal to their legal instruments is always and necessarily an appeal to their power over us. In their capacity to define and enforce rights, states and their agents can always remove those legal instruments or counter them with new ones. | X | X | X | X |  |
| Walker S | 2016 | Some pragmatists dismissing it as idealist and elitist environmentalism (Oosting,2010; Whitford,2009) and more left critics pointing to the limited or problematic race and class makeup of certain alternative food initiatives (Guthman,2008; Holt-Gimenez & Wang,2011), the difficult structural barriers to change faced by grassroots activism (Alkon & Mares,2012; Ghose & Pettygrove,2014), and the ways that garden projects can produce responsiblized neoliberal subjects (Pudup, 2008) and justify state retrenchment (Rosol,2012). | X | X |  | X | X |
| Elmes M, Mendoza-Abarca K, Hersh R | 2016 | three sub-discourses include: - 1) food labour rights with an emphasis on just working conditions and wages for farmworkers and food workers; - 2) slow food with an emphasis on the aesthetic and sustainability dimensions of food and food preparation and consumption; and- 3) food sovereignty (Wittman, 2010; Wittman, Desmarais, & Wiebe, 2010) with an emphasis on food as a basic right, agrarian reform, and sustainable care of natural resources | X | X |  |  | X |
| Sbicca J, Myers J | 2017 | The food justice movement emphasizes the role of race in its critique of, and solutions to, problems in the food system (Allen 2010; Alkon and Agyeman 2011; Holt-Gimenez and Wang 2011). The movement focuses on where food justice emerges, who articulates food justice and why groups demand justice (Cadieux and Slocum 2015; Gottlieb and Joshi 2010). Organizing prioritizes equitable distribution of resources and burdens, the rights of indigenous, low-income communities and communities of colour to a stake in decision-making and control of their food systems, and the dignity and economic rights of food chain workers (Bradley and Herrera 2015; Holt Gimenez and Shattuck 2011). Racial equity, then, requires empowerment-based social change that directly confronts cultural, political, and economic marginalization. | X | X | X | X |  |
| Barron, J | 2017 | Food justice strives to transform systems of privilege and power by addressing injustices that the mainstream food movement (and the alternative food movement), with its focus on producer needs, environmental sustainability and individual consumer choice, has failed to address (Guthman2008, Alkon and Norgaard 2009). | X | X | X | X | X |
| Mello C, King L, Adams I | 2017 | Food justice movements view food as a human right and strive to place communities in charge of their own solutions by providing them with the tools to address the disparities within our food systems and within society at large Holt-Gimenez, Patel, and Shattuck (2009, p 159). | X | X |  | X |  |
| Poulsen M | 2017 | Addressing racial and economic inequities requires the cultivation of food justice. Without an explicit focus on justice, some scholars worry that AFIs (alternative agri-food institutions) may simply serve to placate the privileged, leading to a two-tiered food system in which the non-privileged must cope with the problems created by the industrial food system (Allen 2008; Guthman 2008). Incorporating strategies to improve affordability (e.g., through government entitlement programs) does little to diversify participation if AFIs remain geographically segregated. | X | X |  | X |  |
| Leung M, Agaronov A, Entwistle T, Harry L, Sharkey-Buckley J, Freudenberg N | 2017 | The evidence indicates a need for policy initiatives and environmental approaches to achieve food justice, which could ensure equitable access to healthy foods across all communities (Hilmers, Hilmers, & Dave, 2012; Story, Kaphingst, Robinson-O’Brien, & Glanz, 2008). Food justice initiatives address these issues by increasing people’s capacity to address inequities while seeking to change the [food] system as a whole. (Gottlieb & Joshi, 2010, p. 7). | X | X | X |  | X |
| Horst M | 2017 | Food justice prioritizes concerns for equity and focuses on systemic change and the necessity for engaging in political processes (Mares & Alkon, 2011; Wekerle, 2004). Slocum and Cadieux (2015) - (1) acknowledging and confronting historical, collective social trauma and persistent race, gender, and class inequalities, (2) designing exchange mechanisms that build communal reliance and control, (3) creating innovative ways to control, use, share, own, manage and conceive of land, and ecologies in general, that place them outside the speculative market and the rationale of extraction, and (4) pursuing labour relations that guarantee a minimum income and are neither alienating nor dependent on (unpaid) social reproduction by women. Food justice also entails a focus on democratic processes and participation, paying particular attention to the involvement by people who have experienced food injustices. | X | X | X | X |  |
| Horst M, McClintock N, Hoey L | 2017 | One definition of food justice from the Institute for Agriculture and Trade Policy (2012) is the right of communities everywhere to produce, process, dis-tribute, access, and eat good food regardless of race, class, gender, ethnicity, citizenship, ability, religion, or community. We choose this definition because, unlike some, it calls attention to the multiple ways in which socioeconomically disadvantaged groups are affected across the food system. This comprehensive definition implies a need to focus attention on procedural and distributive justice as well as structural change, a theme we focus on in this review essay. | X | X |  |  |  |
| Harper K, Sands C, Horowitz D, Totman M, Maitin M, Rosado J, Colon J, Alger N | 2017 | The food justice movement emerged in the last decade and represented the convergence of insights, issues, and interests from the alternative food movement and the environmental justice movement (Gottlieb 2009, Gottlieb and Joshi 2010, Alkon and Agyeman 2011). What food justice draws from environmental justice is concern with how certain communities lack access to good food and are often exposed to nutritionally vacuous and even debilitating food, owing to spatial practices such as redlining and disinvestment. Food justice takes a broader social justice perspective on considering how unequal access to quality foods interacts with other injustices contributing to poor health such as environmental exposures, epigenetic factors, and stress (Guthman 2011). | X | X |  | X | X |
| Kato Y, McKinney L | 2017 | Food justice discourse emerged in the last decade to address some of these concerns expressed by the AFNs critique by placing more explicit emphasis on structural inequality and injustice relating to food production and consumption. In doing so, some aspects of the new movement borrow from the environmental justice frame (Capek, 1993) to engage the marginal community for their empowerment in establishing food sovereignty (Alkon and Agyeman, 2011) as well as food access. | X | X |  | X | X |
| Tornaghi C | 2017 | One way to achieve food security and justice is to build on the experiences, practices and values of community empowerment and food sovereignty projects within urban agricultural initiatives. (1) expand our understanding of food justice beyond the more familiar race-, gender- and poverty-based approaches, by pointing out how grassroots food-growing initiatives (commercial or otherwise) can merge environmental ethics, land stewardship and socio-economic benefits to ensure a wider availability of good food, they encourage us to develop reflections on the link between food justice and environmental/spatial justice. (2) a focus on the promises that direct forms of engagement with food production hold as possible pathways for empowerment. | X | X | X | X | X |
| Moragues-Faus M | 2017 | Food security is widely acknowledged as a situation that exists when all people, always, have physical, social and economic access to sufficient, safe and nutritious food that meets their dietary needs and food preferences for an active and healthy life (FAO, 2002). This definition appeals to basic notions of equality. In fact, food security is increasingly associated with notions of sustainability and justice, acknowledging that food systems that are environmentally sound but socially unacceptable would not be resilient in the future and vice versa (Garnett and Godfray, 2012; Sonnino et al., 2014). | X | X |  |  | X |
| Kneafsey M, Owen L, Bos E, Broughton K, Lenn artsson M | 2017 | Food justice movements build on the notion of the right to food, and there is already a body of critical scholarship advocating for a rights-based approach to solving food poverty in the UK (Dowler and O'Connor 2012, Caraher and Dowler 2014, Dowler and Lambie-Mumford 2015). As well as considering the policy and political strategies necessary to defend the right to food, advocates of food justice usually argue that communities and citizens (particularly those most marginalised by the current food system) should have a central and fundamental role in tackling food injustice from the ground up. | X | X | X | X |  |
| Porter C | 2017 | Support of community-based food production is a social change strategy used in the U.S. community food movement that is striving for community food security, sustainability, justice, and sovereignty (Broad, 2016; Saul & Curtis, 2013; Winne, 2008, 2010). Supporting food gardening and other forms of community food production may take society a step closer toward food justice and food security, including because that enables consumers to also become producers (Allen, 1999) | X | X |  | X |  |
| Nunes R | 2017 | Cadieux and Slocum (2015) note that, while the principles of justice may be gaining recognition, it remains unclear whether those who claim to be practicing food justice are doing anything fundamentally different. This problem may be attributed to what scholars, activists, and policymakers have identified as socially just ways of working toward equitable food systems. Moreover, food justice advocates argue that the current global neoliberal industrial food system is broken and challenge its ideological and market dominance |  |  | X | X |  |
| Burga H, Stoscheck C | 2017 | Food justice generally centres on social, environmental, and economic justice (Hayes and Carborne, 2015), and is achieved when there is a food system that serves the community and builds wealth and power for historically disadvantaged groups (Pothu-kuchi, 2015, p. 430). Ultimately, food justice brings social justice into the sustainability movement (Alkon and Norgaard, 2009). The term food justice still has multiple claims, some of which affirm the need for structural change, resource redistribution and democratic processes in the food system, and others that dilute the term by focusing too narrowly on food access in particular, geographic proximity to food sources (Holt-Gimenez and Wang, 2011; Horst, 2017). Horst (2017) asserts that food justice transcends distributive justice and attention to process and acknowledges that food-related injustices are linked to broader socio-economic and power inequities, which in turn are entangled with the legacies of settler colonialism, racialized capitalism, and contemporary neoliberal economic policies (p. 53). Food justice is a progressive response to what Holt-Gimenez calls the corporate food regime a system financed by industrial agribusiness and managed by political actors who create free trade, labour, and property policies that foster conditions needed to enforce a globalized food regime that primarily benefits corporations (Holt-Gimenez and Wang 2011). | X | X | X | X | X |
| Pugh M | 2017 | Food justice is "justice for all in the food system, whether producers, farmworkers, processors, workers, eaters, or communities." (Gottlieb, Joshi 2010). Food justice will require collaboration and commitment from different issue areas. Food production affects the environment employment, and immigration of farmworkers, housing for migrant workers, as well as general rural development issues such as land and community planning in response to increasing urbanization. There are also consumer issues such as public health and food access equality. Food justice even includes business issues such as antitrust, adhesion contracts, and over-regulation of small businesses. These issues affect each other and moving the system toward justice on all sides requires adopting a systemic approach. | X | X | X | X | X |
| Siegner A, Sowerwine J, Acey C | 2018 | Food justice is "justice for all in the food system, whether producers, farmworkers, processors, workers, eaters, or communities." (Gottlieb, Joshi 2010). Horst et al. [Horst 2017] would argue that explicit commitment to food justice and an equity lens is needed for policymakers and planners to create Urban Agriculture spaces that benefit low income and minority communities equally if not more than already advantaged groups [Horst 2017]. | X | X | X | X |  |
| Blake, M | 2018 | Food injustice is defined as an inherent unfairness in the way some aspect(s) of the food system is configured such that some eaters are systematically disadvantaged in their ability to access and/or contribute to the functioning of the food system in which they seek to participate (see also Dixon 2014). | X | X |  | X |  |
| Mello C | 2018 | Alkon and Agyeman (2011) - food justice entails communities of colour providing food for themselves as they imagine new ecological and social relationships. An effective food justice movement requires an analysis that recognizes the food system as a racial project and problematizes the influence of race and class on the production, distribution, and consumption of food. Food insecurity is viewed as one manifestation of structural inequality and racism, especially that due to neoliberal governance. Solution-based activities focus on improving the health outcomes of individuals through educating participants on the interrelationship of locally grown food with sustainable land use practices, economics, and politics. | X | X | X | X | X |
| Glennie C, Alkon A | 2018 | Hislop's (2014) definition Food Justice is the struggle against racism, exploitation, and oppression taking place within the food system that addresses inequality root causes both within and beyond the food chain. Food justice encompasses many issues, including the opportunity to grow or purchase healthy food, diet related health disparities, access to land, and wages and working conditions in agriculture, food processing and restaurant work. But despite these varying topical areas, food justice scholarship is united by a focus on what Julian Agyeman (2013) calls just sustainability, meaning the fusion of concerns for ecological sustainability and social justice. | X | X | X |  | X |
| Swords A, Frith A, Lapp J | 2018 | Food justice brings together individuals and organizations that advocate for food security and sustainable agriculture (Levkoe, 2006). While distinct, they all share basic concerns with equity, food access, sustainability, and health (see Alkon & Norgaard, 2009; Broad, 2016; Gottlieb & Joshi, 2010; Holt-Gimenez, 2010; Winne, 2008). | X | X |  |  | X |
| Pettygrove M, Ghose R | 2018 | Activities associated with pursuit of (more) equitable food systems, including, primarily, equitable access to nutritious foods and community control over food production. | X | X |  | X |  |
| Blumber R, Huitzitzilin R, Urdanivia C, Lorio B | 2018 | Slocum, Cadieux, and Blumberg (2016) created a food justice framework with nodes or entry points, that broadly cover equity and trauma, land, exchange, and labour (see Table 1). Each node draws attention to specific areas that need to be addressed in food justice work. | X | X |  |  |  |
| Weissman E | 2018 | Food justice emerges through the efforts of activists and academics to struggle against the food systems inherent inequalities and to pose a challenge to the dominant white, middle class vote with your fork narrative of the mainstream food movement. Food justice is focused on understanding and challenging structural inequalities. | X | X |  | X |  |
| Croog R, Hayes-Conroy A, Gutierrez-Velez V, Montoya A, | 2018 | Our collaborations have allowed us to see multiple sides of food justice food as an organizing tool and food as nutrition, food as emotion, and food as a right, food/food production as a desire, and food/food production as an ecological action | X | X |  |  | X |
| Reynolds K, Block D, Bradley K, | 2018 | There are many definitions of food justice, as well as debates about both who should define it, and the extent to which the semantics surrounding activism for social justice in the food system are of priority for on-the-groundwork. (See Alkon and Agyeman, 2011; Gottlieb and Fisher, 1996; Gottlieb and Joshi, 2010; Bradley and Herrera, 2015; Hislop, 2014; Herrera cited in Alkon, 2016; Reynolds and Cohen, 2016; Sbicca 2018). At a fundamental level, many actors engaged in thinking about, and undertaking actions for food justice share a concern for uprooting oppression, with a focus on dismantling uneven power dynamics in the food system, from community- to global scales | X |  | X | X |  |
| Porter C | 2018 | Organizers and organizations in the U.S. food justice movement (Bradley & Herrera, 2016; Sbicca, 2012) extensively employ home and community gardening as part of anti-oppression and other transformational strategies for creating equity, health, sustainability, and/or food sovereignty (Broad, 2016; White, 2011, 2011, 2017; Winne, 2008, 2010). No amount of fresh produce will fix urban America’s food and health gap unless it is accompanied by changes in the structures of ownership and immigration laws and a reversal of the diminished political and economic power of the poor and lower working-class (Holt Gimenez & Shattuck, 2011, p. 133). | X | X | X | X |  |
| Gaechter L, Porter C | 2018 | The food justice movement is a budding social movement premised on ideologies that critique the structural oppression responsible for many injustices throughout the agri-food system (Sbicca, 2012, p. 455) | X |  | X | X |  |
| Porter C | 2018 | The body of empirical literature about community-led food security or justice work has been growing; though, it is arguably still short of being proportional to the problems that the work is tackling. Much of this research has been bounded by a focus on one activity, campaign, or project, as opposed to on the work of a community organization (which would be doing multiple such activities, campaigns, and/or projects over time and with paid organizing staff). |  | X |  | X |  |
| Boden S, Hoover B, College M | 2018 | Food Justice is defined by Gottlieb and Joshi (2013) in distributive terms: that the benefits and risks of where, what, and how food is grown and produced, transported, and distributed, and accessed and eaten are shared fairly (p. 6). Both the distributive and evaluative components of food justice must work in tandem to form a food justice movement (Allen, 2010) | X | X |  | X |  |
| Smith B | 2019 | Robert Gottlieb and Anupama Joshi (2010), food justice seeks to achieve equity and fairness in relation to food system impacts and a different, more just, and sustainable way for food to be grown, produced, made accessible, and eaten (p. 223). Alkon and Julian Agyeman (2011) argue that essential to food justice is an analysis that recognizes the food system as a racial project and problematizes the influence of race and class on the production, distribution, and consumption of food (p. 5). | X | X | X |  |  |
| Beriss, D | 2019 | Food justice usually refers to questions of access to healthy and fresh food, along with an under-standing of race and class issues that can impact this access, whereas food sovereignty is a concept developed to focus attention on the work of small-scale food producers in a world dominated by large-scale agribusiness (Mares 2014, p. 35). | X | X |  |  |  |
| Cachelin A, Ivkovich L, Jensen P, Neild M | 2019 | While food access and nutrition are often identified as primary concerns for marginalised communities and there as on for food insecurity and food-related illness, critical food justice scholars use a more expansive lens to suggest a democratised food system is needed, and that solutions based solely in access to healthy food can undermine more systemic approaches. A food sovereignty approach that values the importance of multicultural foodways is an essential element of food justice. | X | X | X | X |  |
| Alkon A, Cadji Y, Moore F | 2019 | How to create food justice and development without displacement due to gentrification. (Cadji and Alkon 2014; Alkon and Cadji 2018) | X |  |  |  |  |
| Smith B | 2019 | Food justice is defined as a historical set of ideological commitments, frameworks, and strategies designed to eradicate inequalities of race, class, gender, and sexuality reproduced in the food system and society that contribute to the rise of hunger, poverty, and food insecurity (Glennie & Alkon, 2018; Hislop, 2015; Sbicca, 2018). | X | X |  |  |  |
| Davenport S, Mishtal J | 2019 | Food Justice is a framework which links food insecurity and food systems to racial, economic, and political inequality (Alkon and Agyeman2011), and a perspective that has an environmental justice focus (Gottlieb 2009) | X | X |  |  | X |
| Gordon C, Hunt K | 2019 | Although food access, policy reform, and both ethical and equitable consumption are important food system concerns, a food justice perspective urges us not to approach these issues in a vacuum. In other words, food politics spans well beyond food itself connecting social and environmental justice. Emphasizing not only the need for more just outcomes, but equities in participatory pro-cesses of advocacy, food justice posits intersectional thinking about the raced, classed, gendered, anthropocentric, and colonial dimensions of food systems | X | X | X | X |  |
| Thompson M, Cockrane A, Hopma J | 2020 | Gottlieb and Joshi define food justice as: ensuring that the benefits and risks of where, what, and how food is grown and produced, transported, and distributed, and accessed and eaten are shared fairly­. It has three key components: seeking to challenge and restructure the dominant food system; a focus on equity and disparities and the struggles by those who are most vulnerable; and establishing linkages and common goals with other forms of social justice activism and advocacy. However, despite its powerful appeal, they argue it remains a relatively unformed concept, subject to multiple interpretations– awork in progress, residing at the edges of an emerging alternative food movement. Therefore, arguably there is a lack of clarity in the food justice movement about what, exactly, food justice is, and what it should look like. It does go beyond food security to connect food issues to fundamental matters of social justice, such as civil rights, environmental justice, health, and poverty. | X | X | X | X | X |
| Siegner A, Acey C, Sowerwine J | 2020 | Urban Agriculture is much more than the production and marketing of food in the city and includes important justice elements (Agyeman and McEntee 2014; Alkon and Agyeman 2011; Alkon and Norgaard 2009). Agroecology places a strong emphasis on human and social values, such as dignity, equity, inclusion, and justice contributing to improved livelihoods of [urban] communities (FAO: element 9). | X |  | X | X |  |
| Thompson D, Johnson K, Cistrunk K, Vancil-Leap A, Nyatta T, Hossfeld L, Mandez G, Jones C | 2020 | Food justice focuses on food access and availability in low-income communities of color as a response to the whiteness of community food security approaches (arose in the 1990s) and its privileging of producer’s needs (Alkon and Guthman 2017: 8). | X | X |  |  |  |
| Aptekar S, Myers J | 2020 | Recent scholarship has underscored that there are strong race and class inequities within the urban agriculture movement in New York City and beyond. Outcomes at the individual, organisational, neighbourhood and city level. Significant differences in how the two sets of community gardeners conceptualize the purpose of their gardens, particularly in constructing them as green spaces, agricultural production sites, and tools for achieving food justice. These differences can be best understood at the intersection of the personal histories of individuals, the organizational settings in which the gardens are embedded, and each neighbourhood’s history of urban renewal and gentrification. Findings show why some community gardens in food insecure communities adopt a food justice vision, while others do not, and how gentrification can amplify racial and class tensions within community gardens and between gardeners and nongardener | X | X |  | X |  |
| Alkon A, Bowen S, Kato Y, Young K | 2020 | Food justice perspective, which examines how racial capitalism affects the production, distribution, and consumption of food. Food justice researchers emphasize the structural conditions that affect food consumption. A food justice framework argues that food is more than just calories and grocery stores. Residential segregation and gentrification, racism in public health and medical institutions and labour conditions through-out the food sector contribute to racial and economic food-related health disparities | X | X |  |  |  |
| Alkon A, Cadji J. | 2020 | Gentrification | X |  |  |  |  |
| Maughan, C., Anderson, C. R., & Kneafsey, M | 2020 | Food justice emphasizes how just sustainability (Agyeman, 2013) cannot be achieved without simultaneously challenging how unequal power relations such as issues of land ownership, labour exploitation, environmental and social injustices (amongst others) play a central role in organizing the production, distribution, and consumption of food (Alkon & Norgaard, 2009). | X | X | X | X | X |
| Sandover R | 2020 | In the food movement, action for food change takes many forms, from street protests to the quiet sustainability of community gardening, to advocacy and acting for food policy change [30,33]. Kneafsey, Owen et al. [30] (p. 622) explore the quiet radicalism of food growing mentorship programmes: we interpret them as examples of capacity building for food justice, through quiet steps rather than radical transformation. |  |  | X | X | X |
| Nicol P, Taherzadeh A | 2020 | From a rights-based approach, food justice is understood as communities exercising their right to grow, sell and eat healthy food [7]. Integrating these understandings of what constitutes a sustainable and just food system, we draw upon the concept of just sustainabilities defined as the need to ensure a better quality of life for all, now and into the future, in a just and equitable manner, whilst living within the limits of supporting ecosystems [8] (p. 5). Just sustainabilities highlights the need to consider the well-being of future generations in any understanding of sustainable food justice, attending not only to inter-generational justice, but also intra-generational justice. Within any conceptualisation of what counts as a just food system, epistemic and contributive justice as well as distributive justice need to be considered [9,10]. | X | X | X |  | X |
| Porter C, Ashcraft C | 2020 | The food justice movement advocates for dismantling institutional racism and policies and programs that support inequalities in the food system (Horst, 2017). | X | X | X | X |  |
| Coulson H, Milbourne P | 2020 | Food justice activism in the US has placed racial equity and racial justice (rooted in civil rights and environmental justice struggles) at the heart of its praxis (critical race theory) (Alkon and Norgaard 2009; Alkon and Agyeman 2011; Myers and Sbicca 2015; Reynolds and Cohen 2016; Penniman 2018). The notion of food justice has increasingly been evoked by various civil society groups as a powerful mobilising concept in the United Kingdom (UK) context. Indeed, the ways in which food justice is deployed in the UK have evaded critical scrutiny (however, see Tornaghi 2016; Kneafsey et al. 2017; Herman and Goodman 2018; Mama D and Anderson 2018), particularly the complex translation politics of drawing upon a concept that has deep situated roots in environmental and social justice movements of the United States (US). This raises the potential issue of mislabelling food-related activities that neglect addressing class and racial injustice (Slocum 2018) or stretching the concept to empty signifier status when applied to different contexts (Heynen et al. 2012), which can ultimately depoliticise activism and stymie marginalised voices. | X |  | X | X | X |
| Noll S, Murdock E | 2020 | Food security generally focuses on ensuring that people have economic and physical access to safe and nutritious food, while food sovereignty (or food justice) movements prioritize the right of people and communities to determine their agricultural policies and food cultures. As food sovereignty movements grew out of critiques of food security initiatives, they are often framed as conflicting approaches within the wider literature. with their focus on the distribution of certain material goods and increasing economic access, food security programs largely accept a distributive model of justice. food sovereignty movements largely accept a more holistic justice paradigm that includes a wide range of social concerns and rights claims. | X | X | X |  |  |
